# Assessment of Morphological, Physical, Thermal, and Thermal Conductivity Properties of Polypropylene/Lignosulfonate Blends

**DOI:** 10.3390/ma14030543

**Published:** 2021-01-23

**Authors:** Mariane Schneider, Noriê Finimundi, Maria Podzorova, Petr Pantyukhov, Matheus Poletto

**Affiliations:** 1 Chemical Engineering, Exact Sciences and Engineering, University of Caxias do Sul (UCS), Caxias do Sul 95070-560, Brazil; msschneider@ucs.br (M.S.); nfinimundi@ucs.br (N.F.); 2 Faculty of Trade Economics and Commodity Science, Plekhanov Russian University of Economics, 117997 Moscow, Russia; mariapdz@mail.ru (M.P.); p.pantyukhov@gmail.com (P.P.); 3 Emanuel Institute of Biochemical Physics, Russian Academy of Sciences, 119991 Moscow, Russia; 4 Postgraduate Program in Engineering of Processes and Technologies (PGEPROTEC), Exact Sciences and Engineering, University of Caxias do Sul (UCS), Caxias do Sul 95070-560, Brazil

**Keywords:** blends, polyolefin, characterization

## Abstract

Lignosulfonate is a cheap material available in large quantities obtained as a byproduct of paper and cellulose. In this work, blends of polypropylene (PP) and sodium lignosulfonate (LGNa) were developed to evaluate the potential use of lignosulfonate as a lightweight, thermal insulation and flame retardant material. The blends were obtained by mixing in a torque rheometer and molded after compression. The blend proprieties were evaluated by physical, morphological, thermal, thermal conductivity, and flammability tests. The measured values were compared with theoretical models. The results indicated that a heterogeneous blend with a higher number of separated domains is formed when the LGNa content increases from 10 to 40 wt%. In addition, the density and thermal conductivity coefficient of the blends studied are not affected by the addition of LGNa. However, when the LGNa content in the blend exceeds 20 wt% the thermal stability and flame retardant proprieties are considerably reduced. The theoretical models based on the rule of mixtures showed a good agreement with the experimental values obtained from blend density, thermal conductivity, and thermal stability. In general, lignosulfonate tested in this work shows potential to be used as a reactive component in polymer blends.

## 1. Introduction

Based on environmental and social concerns such as climate change, global warming, and the growing population in urban areas, government environmental regulations have been encouraging industries to replace inorganic materials by environmentally friendly alternatives [1,2]. The consolidation of the circular economy and the usage of renewable materials can contribute to a more sustainable future [2]. Lightweight materials are an alternative to reduce the fuel consumption in vehicles and can also be used in other engineering fields, such as aerospace applications and construction industry [1,2,3].

Lignin is the second most abundant natural polymer [4,5]. The lignin content in biomass normally ranged from 10–35% [4]. Lignin from hardwood or softwood is a byproduct obtained during paper production [5,6]. As a natural and renewable material, obtained at an affordable cost, lignin and lignin derivates presented potential to be used as a substitute of inorganic reinforcements in polymeric materials [4,5,6]. Due to its complex structure formed by several aromatic rings, lignin is a three-dimensional and highly cross-linked macromolecule which has interesting chemical and physical properties [7,8].

Lignin and its derivative products can be obtained from the cellulose and paper industries. The two pulping processes vastly used in the paper industry are the sulfite and sulfate (Kraft) process [9,10]. Lignosulfonate is a lignin derivate obtained as a by-product in the sulfite process while Kraft lignin is obtained from the sulfate process [10]. Lignosulfonates have a higher molecular weight and higher sulfur content than Kraft lignin [9,10,11]. Budin and Schoenmakers [9] related that only 2% of the lignin by-products which are obtained by the pulp and paper industries are used commercially [9]. Lignin and lignin derivate products are available in relatively large quantities with a low price [12,13]. The industrial applications of lignosulfonates included low-value products, such as binders and dispersing agents [12,13].

Huang et al. [14] developed biodegradable plastics from mixtures of soy protein isolated and lignosulfonate using glycerol as a plasticizer [14]. The blends were obtained by compression molding. The results indicated that the lignosulfonates content from 30 to 40 parts in the blends improve both the tensile strength and Young’s modulus of soy protein alone. The authors also related the lower water absorption from the blends when compared with soy protein. Lin et al. [15] developed a blend of calcium lignosulfonate and biodegradable poly(butylene succinate) (PBS) molded by compression [15]. The authors also observed an increase in the blend rigidity resulting in higher Young’s modulus values for blends with 30–50 wt% of calcium lignosulfonate when compared with PBS. Wang et al. (2016) used maleated lignosulfonate (MLS) produced by esterification with maleic anhydride and unmodified lignosulfonate incorporated into poly(e-caprolactone) (PCL) via melt-blending [16]. The authors also observed an enhancement in the mechanical properties of the blends in comparison with PCL.

As reported in previous studies, the addition of lignosulfonate in polymers can improve the mechanical properties of the blend. This behavior may be associated with the higher rigidity of the lignosulfonate based on its complex chemical structure formed by several aromatic rings in a three dimensional and highly cross-linked macromolecule, similar to the lignin structure. However, numerous works previously evaluated the mechanical properties of polymer lignosulfonate blends. Therefore, the literature lacks studies about other properties of polymer lignosulfonate blends and the usage of these blends to develop lightweight materials that can be used by the construction industries. In this way, this work focuses on the development and characterization of PP/sodium lignosulfonate blends that can be used as office partition panels and wall cladding. The morphological, physical, thermogravimetric, thermal conductivity, and flammability properties of the blends were evaluated and compared with theoretical results.

## 2. Materials and Methods

Polypropylene homopolymer, grade H103, supplied by Braskem (São Paulo, Brazil) with a density of 0.905 g/cm^3^ and melt flow index of 40 g/10 min (230 °C/2.16 kg) was used. Sodium lignosufonate with a commercial name of Ultrazine NA with a mean particle size of 5 µm from Borregaard Linotech Brazil was also used to develop the blend.

### 2.1. Blend Production

A Haake torque rheometer was used to the mixture of PP and LGNa. Both components were previously oven-dried at 80 °C for 4 h. The rotation speed used during the mixture was 60 rpm and occurs at 180 °C. PP was firstly added in the torque rheometer and after its melting LGNa was added. After the LGNa addition, the mixture was processed for 3 min to better homogenize the polymer blend. The blends were prepared with 10–40 wt% of LGNa.

The polymer blends were compression molded in sheets with a thickness of 3.2 mm using a hot press operating at 180 °C for 3 min at 5 tons.

### 2.2. Blend Characterization

The blend morphology was investigated using scanning electron microscopy in a FEG SEM Tescan Mira 3 (Brno, Czech Republic) at 15 kV. The samples were sputter-coated with gold before examination.

The density of PP and its blends with LGNa were determined in triplicate experiments according to ASTM D792 from the compression molded samples with 2 × 2 cm^2^. The void content in the blends were obtained according to ASTM D 2734. The LGNa density was determined in triplicate experiments using a pycnometer based on the ASTM D297 method. The sample was oven-dried at 105 °C for 48 h before the test.

The thermal conductivity of PP and its blends with LGNa was obtained in triplicate experiments according to ISO 8302 using the guarded hot plate method. The theoretical models proposed by Bruggeman [17], Botcher [17], and De Loor [17], Equations (1)–(3), respectively, were used for the prediction of the thermal conductivity of PP/LGNa blends.

K_b_ = K_m_/(1 − Φ)^3^ (1)

K_b_ = K_m_/(1 − Φ) (2)

K_b_ = [K_m_ (1 + Φ)]/(1 − 2Φ) (3)

where K_b_ and K_m_ are the thermal conductivity of the blend and continuous phase, respectively. Φ is the volume fraction of the particles.

The thermogravimetric analyses (TGA) of the samples were carried out using a TGA-50 (Shimadzu, Kyoto, Japan) in a nitrogen atmosphere at a gas flow rate of 50 mL/min. A mass sample of approximately 10 mg was used. The temperature range used and heating rate adopted were 23–800 °C and 10 °C/min, respectively.

The burning characteristics of PP and its blends with LGNa were evaluated according to the flammability vertical test proposed in the UL 94 standard and ASTM D3801. Samples bars with 127 × 12.7 × 3.5 mm^3^ were used. Five specimens of each sample were tested.

## 3. Results and Discussion

**Figure 1:**
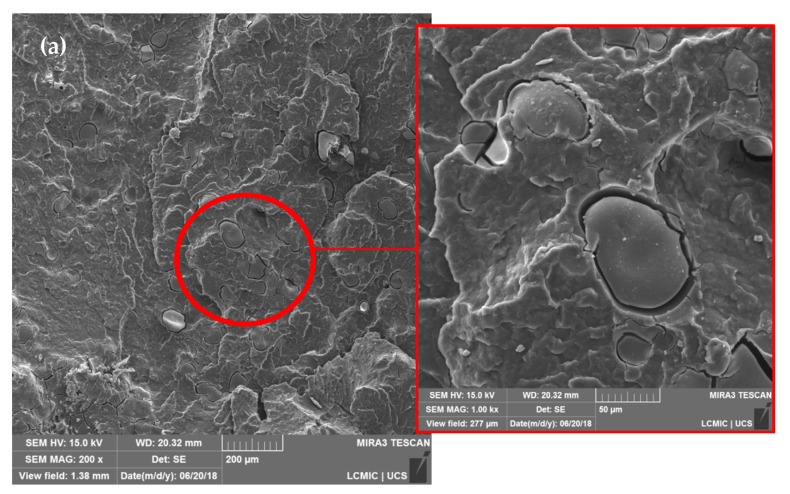

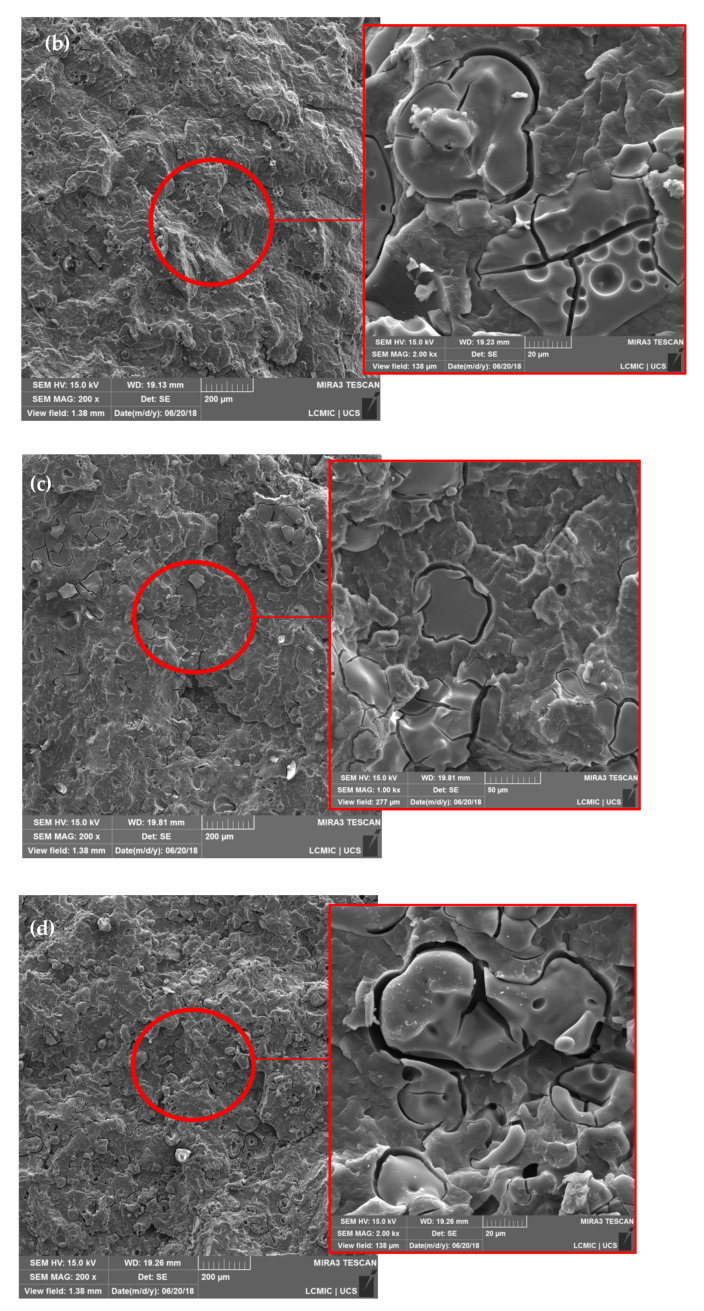
SEM micrographs of polypropylene/sodium lignosulfonate (PP/LGNa) blends with 10 (**a**), 20 (**b**), 30 (**c**), and 40 wt% (**d**) of LGNa with magnification of 200× and detail with magnification of 1000×.

The morphological characteristics of the studied blends can be observed in Figure 1.

In general, the lignosulfonate particles are spherical and randomly dispersed in PP without the formation of particle agglomerates. As can be seen in detail in Figure 1, there is a low interfacial adhesion between the PP and LGNa particles. This behavior can be explained by the incompatibility between the hydrophobic PP and higher hydrophilic characteristics of lignosulfonate probably due to the oxygen groups presented on the surface of LGNa. Lignosulfonates are compounds with hydrophobic phenylpropane units and strong hydrophilic sulfonic groups present on the surface of the particles [13,18]. Therefore, the weak interactions between the hydrophobic PP and the polar groups of the LGNa result in the formation of a heterogeneous blend with large dispersed LGNa particles. Cazacu et al. [19] also observed phase separation and an increase in the dimensions of separated domains with increases in the ammonium lignosulfonate content on the ethylene-propylene copolymer blend [19].

**Figure 2:**
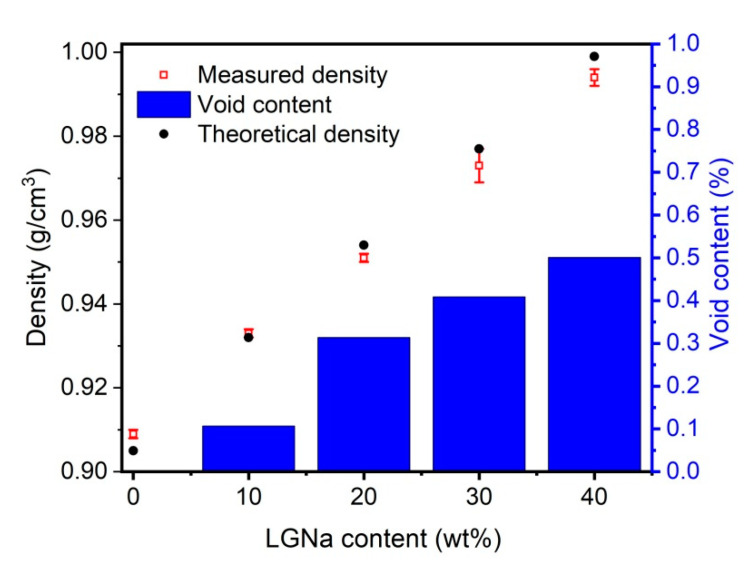
Theoretical and measured density of PP and blends of PP/LGNa showing the void content of all the blends studied.

The density of LGNa obtained by the pycnometer method was 1.135 ± 0.003 g/cm^3^. The lignin density normally ranges from 1.35 to 1.50 g/cm^3^ [20]. The blends theoretical density was determined based on the rule of mixtures and compared with the experimental values [21]. Figure 2 shows the density values for the measured and theoretical density of the blends studied.

The LGNa addition from 10 to 40 wt% linearly increased the blend density, as shown in Figure 2. However, even with the addition of 40 wt% of lignosulfonate the blend density increased only 10% when compared with pristine PP. This result indicates that the LGNa addition in PP generates a lightweight material that has potential to be in construction industries. The theoretical density values are very similar to the measured values which indicate that the rule of the mixture can be used to estimate the blend density. The void content increases with the LGNa content in the blend, as shown in Figure 2. This is an expected behavior based on the SEM results presented in Figure 1. As the LGNa content increases, a more pronounced phase separation may occur which results in higher gaps between both phases resulting in a higher void content.

**Figure 3:**
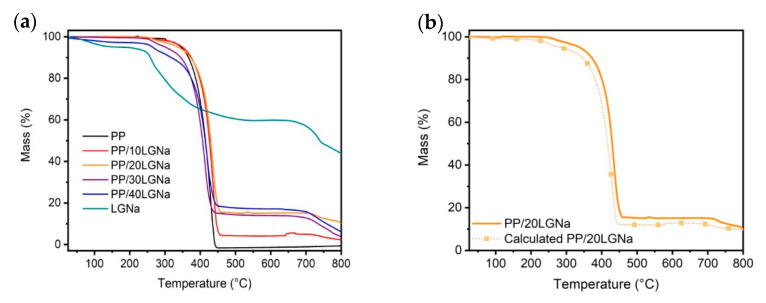
Thermogravimetric curves of PP, LGNa, and its blends (**a**) and comparison of the expected and experimental thermogravimetric curves of the PP/LGNa blend with 20 wt% of LGNa (**b**).

The thermogravimetric results of PP and LGNa particles show distinct processes of weight loss occurring at different temperatures, as shown in Figure 3a. For PP, there is only one degradation step associated with the random cleave of C-C bonds, with a higher rate of weight loss temperature (T_max_) at 432 °C, while LGNa particles show three degradation steps. The first step of weight loss occur between 50–150 °C, with a peak loss rate centered at 100 °C, and can be associated with the absorbed moisture [22]. From the thermogravimetric curve in Figure 3a, it is possible to observe that LGNa starts a more pronounced degradation process at around 220 °C extending to approximately 380 °C, with a T_max_ centered at 267 °C. The decomposition of oxygen-containing groups and cleave of C-C bonds occur in this degradation step [22,23]. Some aromatic compounds presented in the lignosulfonate structure such as phenol, guaiacol or syringol, alkyls, some sulfur- and/or sodium-containing small molecules were released forming different carbon structures [23]. A last weight loss occurs between 650–750 °C and might be mainly attributed to the reactions between carbon and sodium-containing inorganic salts associated with the decomposition of residual unstable oxygen containing groups and carbon groups [23].

The PP/LGNa blends exhibited two main degradation steps. The first one is attributed to the main degradation of oxygen-containing groups and cleaves of C-C bonds in LGNa, as previously explained, and occurs approximately at the same T_max_ observed by LGNa particles. However, the T_max_ associated with the PP degradation firstly increases with the addition of 10 and 20 wt% of LGNa, but decreases with the addition of 30 and 40 wt% of lignosulfonate, as presented in Table 1. The higher number of separated domains formed when higher quantities of LGNa particles were added and the incompatibility between both materials might contribute to this result. LGNa has a lower thermal stability than PP. When higher quantities of lignosulfonate particles are added, a decomposition process may start at lower temperatures reducing the blend thermal stability. The initial weight loss temperature (T_i_) considered, in this work, when the sample loses 3 wt% of its initial mass also corroborate these results, as presented in Table 1. The T_i_ values considerably decrease as the lignosulfonate content increases in the blend formulation.

The thermogravimetric results obtained from the blends were compared to the calculated curves obtained from the weighted mean of pristine PP and LGNa particles, as shown in Figure 3b. The calculated curve is obtained based on the rule of mixtures as previously proposed by Araújo et al. [24]. In theory, the blends whose curves were located below the calculated curve are less stable than expected, showing antagonistic interactions [24]. On the other hand, the blends whose curves are located above the calculated curve are more stable than expected, showing synergistic interactions [24]. The addition of lignosulfonate promotes a synergic effect on the blends thermal stability with 10–30 wt% of LGNa, as can be seen for the example of the blend with 20 wt% of LGNa in Figure 3b. The calculated T_i_ values presented in Table 1 for blends with 10–30 wt% of LGNa are lower than the T_i_ values obtained from experimental thermogravimetric curves. This result indicates synergistic interactions between PP and LGNa particles which are responsible for increasing the blend thermal stability. However, when the LGNa content reaches 40 wt%, antagonistic interactions between PP and LGNa occurs. One possible explanation for this is the fact that the blend with 40 wt% of LGNa presents the highest number of separated domains and also the highest content of oxygen groups at the lignosulfonate surface, which may accelerate the degradation process, reducing the blend thermal stability.

**Figure 4:**
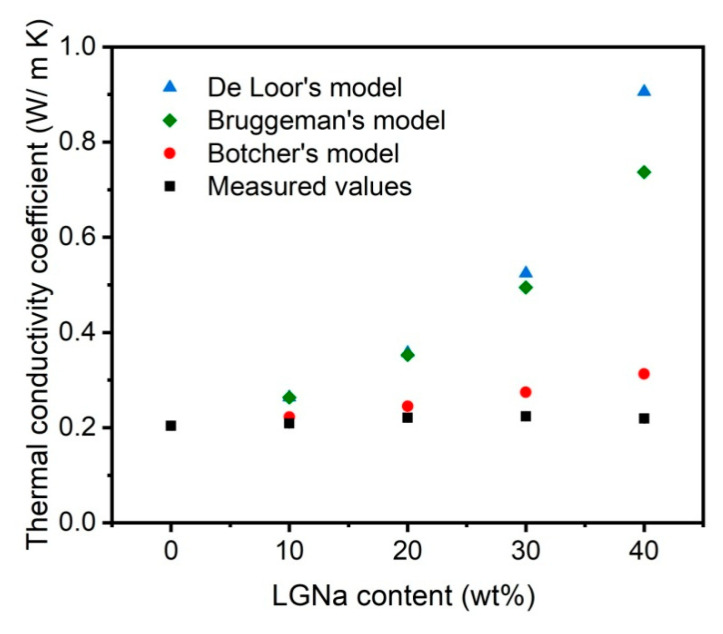
Thermal conductivity coefficients for PP with different contents of LGNa compared with theoretical models.

Thermal conductivity can be defined as the material’s ability to molecularly transport heat through conduction [25,26,27]. In polymeric materials, the heat conduction occurs mainly by the vibration and rotation of chain atoms, which can promote the motion of polymer chains [25,28]. The thermal conductivity coefficient (*k*) in thermoplastic composites is affected by the chain structure and orientation, interchain interactions, and crystallinity [25]. When another material is blended with a thermoplastic polymer these parameters may be changed and directly affect the thermal conductivity of the resulting material. On the other hand, thermal conductivity is an important parameter to be considered for the thermal comfort of buildings [25,29]. Figure 4 shows the thermal conductivity coefficients measured for PP and its blends with LGNa.

The measured thermal conductivity coefficient of PP was 0.204 ± 0.003 W/m K. PP is a semi-crystalline polymer that can exhibit *k* values between 0.10–0.25 W/m K [25], depending on its crystallinity degree. Ebadi-Dehaghani et al. [17] obtained a *k* value of 0.22 ± 0.04 W/m K using a guarded heat flow meter method [17]. The addition of LGNa particles did not considerably affect the thermal insulating nature of the PP. The *k* values from the PP/LGNa blends ranged from 0.209–0.224 W/m K. Considering the atomic structure of the blends, the main energy transfer occurs by the transport of phonons through the polymer structure, since the free movement of electrons is not possible [25]. The LGNa particles may act as barriers to energy transfer through the PP structure and the separate domains formed due to the incompatibility between PP and LGNa might contribute to maintaining the low thermal conductivity of the blends. In addition, when the LGNa content increased from 30 to 40 wt%, the *k* value reduced from 0.224 ± 0.01 W/m K to 0.219 ± 0.01 W/m, which may indicate that the higher number of separated domains formed with a higher content of LGNa particles, as shown in Figure 1d, contributing to the reduction of the energy transfer through the blend.

In general, the blends developed in this study presented an average density of 950 kg/m^3^ with a mean thermal conductivity of 0.22 W/m K. When compared to some materials traditionally used by construction industries such as the gypsum and cement boards, a thermal conductivity of 0.27 and 0.35 W/m K, respectively, was presented but with a higher density than PP/LGNa blends, ranging from 1033 to 1150 kg/cm^3^ [30].

The experimental *k* values were compared with theoretical values obtained from theoretical models, as shown in Figure 4. Bottcher’s model predicts fairly well the *k* values until 20 wt% of LGNa particles. Bottcher’s model as also Bruggeman’s model assumes that the interactions among the particles can occur according to the increase in the volume fraction of one component, which can directly influence the material thermal conductivity [31,32].

Previous studies related to the potential of lignin and lignin derivates as potential bio-based flame retardants [33,34]. Flammability tests showed that samples with 10 and 20 wt% of LGNa can be classified as the V-2 category according to ASTM D3801, while PP and blends with 30 and 40 wt% of LGNa cannot be classified according to this standard. The obtained results are presented in Table 2.

Samples containing 30 and 40 wt% of LGNa showed greater fluidity during the flammability test when compared with the blends with a lower LGNa content, which favored the total burning of the specimen followed by the flaming drip after the PP flow. The average time for flame extinguishes increases with the LGNa content. Blends containing 10 and 20 wt% of LGNa presented the mean time for flame extinguish values of 3.2 and 7.6 s, and those containing 30 and 40 wt%, expanded to 34 and 45 s, respectively. This behavior indicates that the LGNa content higher than 20 wt% does not contribute to improving the performance of the blend as the flame retardant material. When the LGNa content in the blend is higher than 20 wt% more oxygen groups are in the particle surface which probably enhances the possibility of blend ignition. This behavior is in agreement with the TGA results previously discussed. On the other hand, sodium can react with the degradation products during combustion which may result in the formation of inorganic salts, such as Na_2_CO_3_ and Na_2_SO_4_ [23], and might accelerate the blend thermal decomposition.

## 4. Conclusions

The usage of lignosulfonate in different proportions caused changes in morphological, thermal, thermal conductivity, and flammability properties of the blends studied. The morphological result confirms the formation of a heterogeneous blend with a cracked appearance. The formation of lignosulfonate domains increases with the LGNa content. The blend density and thermal conductivity are not considerably affected by the lignosulfonate addition resulting in a lightweight material with thermal insulation characteristics. The thermogravimetric results indicate that the LGNa addition reduces the blend thermal stability. The LGNa tested in this work has the potential to act as a flame retardant. However, its content in the blend cannot exceed 20 wt%. The results obtained from the PP/LGNa blends evaluated in this study encourage the utilization of lignosulfonate as a cheap, lightweight, thermal insulation, and flame retardant material.

## Figures and Tables

**Table 1:** Temperature parameters evaluated for PP, LGNa, and its blends.

wt% PP/wt% LGNa	T_i_ (3 wt% Weight Loss) (°C)	Calculated T_i_ (3 wt% Weight Loss) (°C)	T_max_ LGNa (°C)	T_max_ PP (°C)
100/0	332	−	−	432
0/100	93	−	267	−
90/10	327	278	267	435
80/20	305	250	266	438
70/30	273	241	268	418
60/40	222	230	266	421

**Table 2:** Flammability properties of PP and its blends with LGNa particles.

Criteria Conditions	PP	90/10	80/20	70/30	60/40
Afterflame time for each individual specimen, *t*1 or *t*2 ^1^	≥30 s	≤30 s	≤30 s	≥30 s	≥30 s
Total afterflame time for any condition set (*t*1 plus *t*2 for the five specimens)	≥250 s	≤250 s	≤250 s	≥250 s	≥250 s
Afterflame plus afterglow time for each individual specimen after the second flame application (*t*2 + *t*3)	≥60 s	≤60 s	≤60 s	≥60 s	≥60 s
Afterflame or afterglow of any specimen up to the holding clamp	Yes	No	No	Yes	Yes
Cotton indicator ignited by flaming particles or drops	Yes	Yes	Yes	Yes	Yes

^1^*t*1 Afterflame time after the first flame impingement, s, of the *i*th specimen; *t*2 afterflame time after the second flame impingement, s, of the *i*th specimen; and *t*3 afterflame and afterglow times after the second flame application.

## Data Availability

The data presented in this study are available on request from the corresponding author.
